# LAY YOUR BURDENS DOWN: DEFERRED RELIGIOUS COPING AS A MARITAL STRESS BUFFER FOR OLDER AFRICAN AMERICAN COUPLES

**DOI:** 10.1093/geroni/igz038.3393

**Published:** 2019-11-08

**Authors:** Antonius D Skipper

**Affiliations:** Winston-Salem State University, Winston-Salem, North Carolina, United States

## Abstract

Although growing bodies of research explore the dynamics of minority families, few consider the African American family from a strengths-focused perspective. Stressors that threaten familial stability, such as financial strain, health disparities, and sporadic employment, disproportionately affect African American families and contribute to high rates of dissolution. In response, African American families often rely on religion as a source of coping and resilience. While existing literature adequately captures the frequency of religious-based responses to stress, opportunities to examine the nuances and underlying processes of religious coping for African American families exist. This study addresses the need to move beyond the broad measures of religiosity and religious coping, in exchange for a more in-depth exploration of how various forms of religious coping, specifically deferred coping, impact well-being. Deferred religious coping is characterized as a complete reliance on a higher power during a time of stress. Thirty-five older African American couples (N=70 individuals), representing 11 states in the U.S., were interviewed regarding the dynamics of deferred religious coping in the marital dyad. Following the digital recording and transcription of the narrative data, the interviews were analyzed with an open coding procedure consistent with grounded theory and Numeric Content Analysis (Marks, 2015). Analyses reveal that nearly 75% of the couples interviewed utilized deferred religious coping in response to stressors that could threaten marital stability. Further, salient themes include: (1) The Three-Party, Divine Triangle of Marriage, (2) Deferring Health Problems Reduces Worry, and (3) A Healthy Work-Family-Prayer Balance. Implications for practice are also discussed.

